# Oropharyngeal swallowing physiology and safety in patients with Idiopathic Pulmonary Fibrosis: a consecutive descriptive case series

**DOI:** 10.1186/s12890-022-02232-3

**Published:** 2022-11-17

**Authors:** Amal Alamer, Rhys Jones, Michael Drinnan, A. John Simpson, Mike Griffin, Joanne M. Patterson, Abdullah Althuwaybi, Chris Ward, Ian A. Forrest

**Affiliations:** 1 Translational and Clinical Research Institute, School of Medical Sciences, Newcastle University, Newcastle Upon Tyne, United Kingdom; 2 Respiratory Care Department, College of Applied Medical Sciences, Imam Abdulrahman Bin Faisal University, Dammam, Kingdom of Saudi Arabia; 3 The James Cook University Hospital, Middlesbrough, United Kingdom; 4 Northern Medical Physics and Clinical Engineering, Newcastle Upon Tyne Hospitals Trust, Newcastle Upon Tyne, United Kingdom; 5 The Royal College of Surgeons of Edinburgh, Edinburgh, United Kingdom; 6 School of Health Sciences, Thompson Yates Building, University of Liverpool, , Liverpool, United Kingdom; 7 Royal Victoria Infirmary, The Newcastle Upon Tyne Hospitals NHS Foundation Trust, Newcastle Upon Tyne, United Kingdom

**Keywords:** Idiopathic Pulmonary Fibrosis, Videofluoroscopy Swallow Study, Oropharyngeal swallowing, The Modified Barium Swallow Impairment Profile, Aspiration, The Eating Assessment tool EAT-10

## Abstract

**Introduction:**

Dysphagia occurs in multiple respiratory pathophysiologies, increasing the risk of pulmonary complications secondary to aspiration. Reflux associated aspiration and a dysregulated lung microbiome is implicated in Idiopathic Pulmonary Fibrosis (IPF), but swallowing dysfunction has not been described. We aimed to explore oropharyngeal swallowing in IPF patients, without known swallowing dysfunction.

**Methods:**

Fourteen consecutive outpatients with a secure diagnosis of IPF were recruited and the 10-item Eating Assessment Tool (Eat 10) used to assess patient perception of swallowing difficulty. Oropharyngeal swallowing was assessed in ten patients using Videofluoroscopy Swallow Studies (VFSS). The studies were rated using validated scales: Penetration-Aspiration Scale (PAS); standardised Modified Barium Swallow Impairment Profile (*MBSImP*).

**Results:**

EAT-10 scores indicated frank swallowing difficulty in 4/14 patients. Videofluoroscopy Studies showed that 3/10 patients had airway penetration, and one aspirated liquid without a cough response. Median *MBSImp* for oral impairment was 5, range [3–7] and pharyngeal impairment 4, range [1–14] indicating, overall mild alteration to swallowing physiology.

**Conclusion:**

We conclude that people with IPF can show a range of swallowing dysfunction, including aspiration into an unprotected airway. To our knowledge, this is the first report on swallowing physiology and safety in IPF. We believe a proportion of this group may be at risk of aspiration. Further work is indicated to fully explore swallowing in this vulnerable group.

## Introduction

Idiopathic pulmonary fibrosis (IPF) is a chronic life-threatening lung disease, with poor prognosis, characterised anatomically by progressive scarring of the lung and symptomatically by exertional dyspnoea, with irreversible loss of pulmonary function [1].

While the aetiology is unknown, emerging data suggest an important association with aspiration [2] linked with gastric reflux. The term aspiration refers either to the direct inhalation of secretions or ingested materials from the oesophagus or from the oropharynx into the airways. Aspiration by either route can lead to the presence of damaging agents into the airways and the lungs, which is thought to drive the onset and/or progression of a broad range of pathological diseases that affect the airways and lungs [3]. To date, investigations of IPF and aspiration have been limited to gastro-oesophageal reflux disease in which gastric content refluxes up into the oesophagus, with the potential to pass into the airway via the pharynx (microaspiration) [4]. Concerns about the potential role of gastro oesophageal reflux-associated aspiration in IPF pathophysiology have been sufficient to prompt an influential multi-centre pilot of fundoplication [5]. A dysregulated lung microbiome has also been implicated in high profile studies [6].

The pharynx is a shared tube for breathing and swallowing. Because of this, normal swallowing is a complex process, coordinated with breathing to protect the airway. Patients with chronic respiratory diseases are at risk of oropharyngeal dysphagia and swallowing difficulties may go unreported [7, 8]. Early evidence suggests 20% of COPD patients have a swallowing impairment, and subclinical aspiration, identified on Videofluoroscopy Swallow Studies (VFSS) [9]. Prior study found 44% of COPD patients reported swallowing difficulties symptoms on a patient-reported outcome measure [7]. The parenchymal lung scarring, and hypoxaemia seen in IPF patients may act to disrupt respiratory-swallow coordination leading to dysfunction. Surprisingly we are unaware of studies investigating swallowing dysfunction in people with IPF. The purposes of this study were to explore the perception of swallowing difficulty and oropharyngeal swallowing physiology in people with IPF. This was carried out in outpatients without known or suspected swallowing problems.

## Material and methods

### Participants

Consecutive outpatients with a secure diagnosis of IPF according to European Respiratory Society/American Thoracic Society were recruited from the regional Interstitial Lung Disease (ILD) clinic at the Royal Victoria Infirmary, Newcastle upon Tyne between March 2014 and February 2015. Outpatients with IPF are reviewed on a regular basis at the ILD clinic at the Royal Victoria Infirmary. Recruits were drawn from within this clinic population. Written informed consent was obtained using a printed form prior to the study assessment. Inclusion criteria included competent adults (over the age of 18) with a secure diagnosis of IPF according to the American Thoracic Society (ATS), European Respiratory Society (ERS), Japanese Respiratory Society (JRS) and Latin American Thoracic Society (LATS) clinical practice guidelines [10]. Accurate diagnosis is also supported by discussions among the Newcastle Hospitals Multi-Disciplinary Team (MDT). Exclusion criteria: pregnancy; neurological disease; dementia; gastro-intestinal disease, excepting controlled reflux symptoms; head & neck pathology excepting tonsillectomy/adenoidectomy; previous thoracic surgery; stroke. The patients were screened and excluded if they had a neurological diagnosis that may be associated with dysphagia. The study was described in details, with full written consent taken at the outset, before study activities commenced. All methods were performed in accordance with the approval guidelines and regulations.

### Swallowing questionnaire

Eating Assessment tool (EAT-10) is a quick, self-administered and widely used validated questionnaire, which can be used to assess dysphagia symptoms [11]. It has been used previously with patients with chronic respiratory disease such as COPD [7, 12]. It consists of 10 questions regarding swallowing difficulty. Each question is scored on a 5 point Likert scale from 0 (no problem) to 4 (severe problem). The total EAT-10 score is calculated by adding up the scores across the 10 statements (highest score = 40). A total score of 3 or more indicates swallowing difficulty [13].

### Swallowing assessment

Swallowing was assessed by a Videofluoroscopy Swallow Study (VFSS). VFSS is a dynamic radiographic examination using fluoroscopy to capture and record real-time bolus flow throughout all stages of swallowing. A Speech and Language Therapist and a radiologist, performed the VFSS examinations. The examination typically includes testing different bolus volumes and constituents [14]. The participants were seated in an upright position. Test boluses were thin liquid (5 and 20 mL); 5 mL paste (custard) and solid (1/4 biscuit) mixed with radiopaque barium sulphate (E-Z-PAQUE), conducted in the lateral plane and one 10 mL liquid bolus with an anterior–posterior view. Liquids were administered first to avoid confounding the results due to remaining residue in the pharynx after ingesting solid consistencies. A penny was taped to the subject’s chin during the swallowing study. The circular shape of the penny minimizes the impact of head rotation and the known diameter of the coin allows for calibration of pixels per cm and thus calculation of areas and displacement on VF.

#### VFSSs analysis

Studies were rated using two validated scales:

1. The standardised Modified Barium Swallow Impairment Profile *(MBSImP),* a tool used to evaluate swallowing efficiency that measures 17 physiological components of adult swallowing mechanism using ordinal scaling. Ratings of 0 to 2, 3, or 4 points *per* component, with each score representing a unique observation of either structural movement, bolus flow or both from the VFSS [14, 15]. It covers 3 functional domains of the swallow; oral (0–22), pharyngeal (0–29) and oesophageal (0–4). The Overall impression (OI) score is the worst and the most impaired score for all bolus amounts and consistencies. The Oral impairment score and Pharyngeal impairment score were calculated by summing up the OI scores [14]. Scores were interpreted according to a clinically validated classification system [16].
2. The Penetration Aspiration Scale (PAS) is a tool used to evaluate swallowing safety, ranging in value from 1 to 8, recording the presence of laryngeal penetration/sub-glottic aspiration (1 = no airway invasion, 2–5 = penetration, 6–8 = aspiration) [17]. Penetration is defined as the passage of food or fluid into the airway just above the level of the vocal cords. Whereas, aspiration is defined as the passage of food or fluid below the level of the vocal cord [18].

Higher scores for both tools indicate poorer swallowing.

## Results

**Fig. 1:**
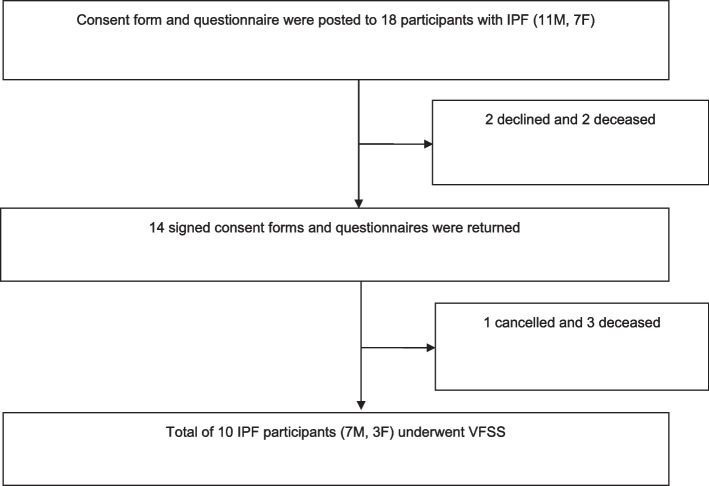
Study flow chart. IPF, Idiopathic pulmonary fibrosis; VFSS, Videofluoroscopy Swallow Study

We recruited 14 participants with IPF: 10 males, 4 females, median: 68 years, range 51–82 years. Ten IPF patients: 7 males, 3 females, median: 63 years, range 51–77 years underwent the VFSS (Fig. 1: Consort flow diagram for study).

### EAT-10: self-reported swallowing symptoms

**Fig. 2:**
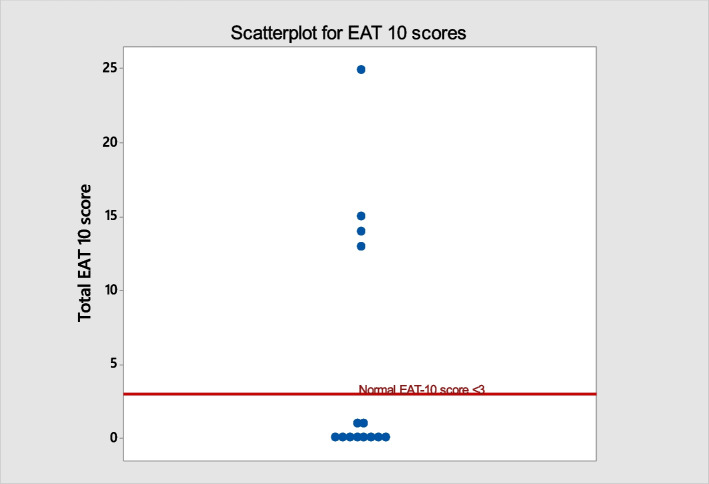
Scatterplot showing: EAT-10 score (y-axis) for IPF patients. The red line shows the upper score for EAT-10. A score ≥ 3 indicates abnormal EAT-10 scores. IPF, Idiopathic pulmonary fibrosis; EAT-10; The Eating Assessment tool EAT-10

A total of 14 participants with IPF completed the EAT-10 questionnaires. All data are presented in Fig. 2. The total median EAT-10 score was 0, range 0–25. Scores were raised in four patients, with values of 25, 15, 14 and 13. These exceeded the normal cut off < 3, indicating swallowing difficulty [13].

### Swallowing physiology and safety

#### The clinical characteristics

The clinical characteristics for the IPF patients underwent VFSS are presented in Table 1. Overall, there were 10 IPF participants who completed the VFSS, the median age of participants was 63 years [range: 51–77]. Most participants were males (7/10, 70%) and ex-smokers (7/10, 70%). The median Body Mass Index (BMI) was 26.2 kg/m2 [range: 20.3–45.6]. The median modified Medical Research Council dyspnoea scale (mMRC) was 2 [range: 1–4]. The median FEV1/FVC% was 80.5% [range: 74–93] and the median FVC % of predicted was 72% [range: 51–92] indicating a restrictive lung pattern [19]. The median TLCO % of predicted was 60% [range: 27–78], showing impaired lung gas transfer.

**Table 1:** The clinical characteristics for the IPF patients underwent Videofluoroscopy Swallow Study (VFSS)

Factor	Value
Number	10
Age,years	63 [51–77]
Sex, Male/Female	7/3
BMI, kg/m2	26.2 [20.3–45.6]
Smoking status: current smoker/ex-smoker	3/7
mMRC	2 [1–4]
FVC % predicted	72 [51–92]
FEV_1_/FVC, %	80.5 [74–93]
TLCO% of predicted	60% [range:27–78]

Data presented as median (ranges), unless otherwise indicated. *BMI* Body mass index, *mMRC* Modified medical research council dyspnoea scale, *FEV1* Forced expiratory volume in 1 s, *FVC* Forced vital capacity, *TLCO* Transfer factor for carbon monoxide

#### The Videofluoroscopy Swallow Study (VFSS)

All data are presented in Table 2. Median *MBSImp* for oral impairment was 5 [range: 3–7] and pharyngeal impairment 4 [range 1–14] indicating, overall mild alteration to swallowing physiology. Patient 1 scored 14 in the pharyngeal impairment indicating mild/moderate impairment [16].

**Table 2:**
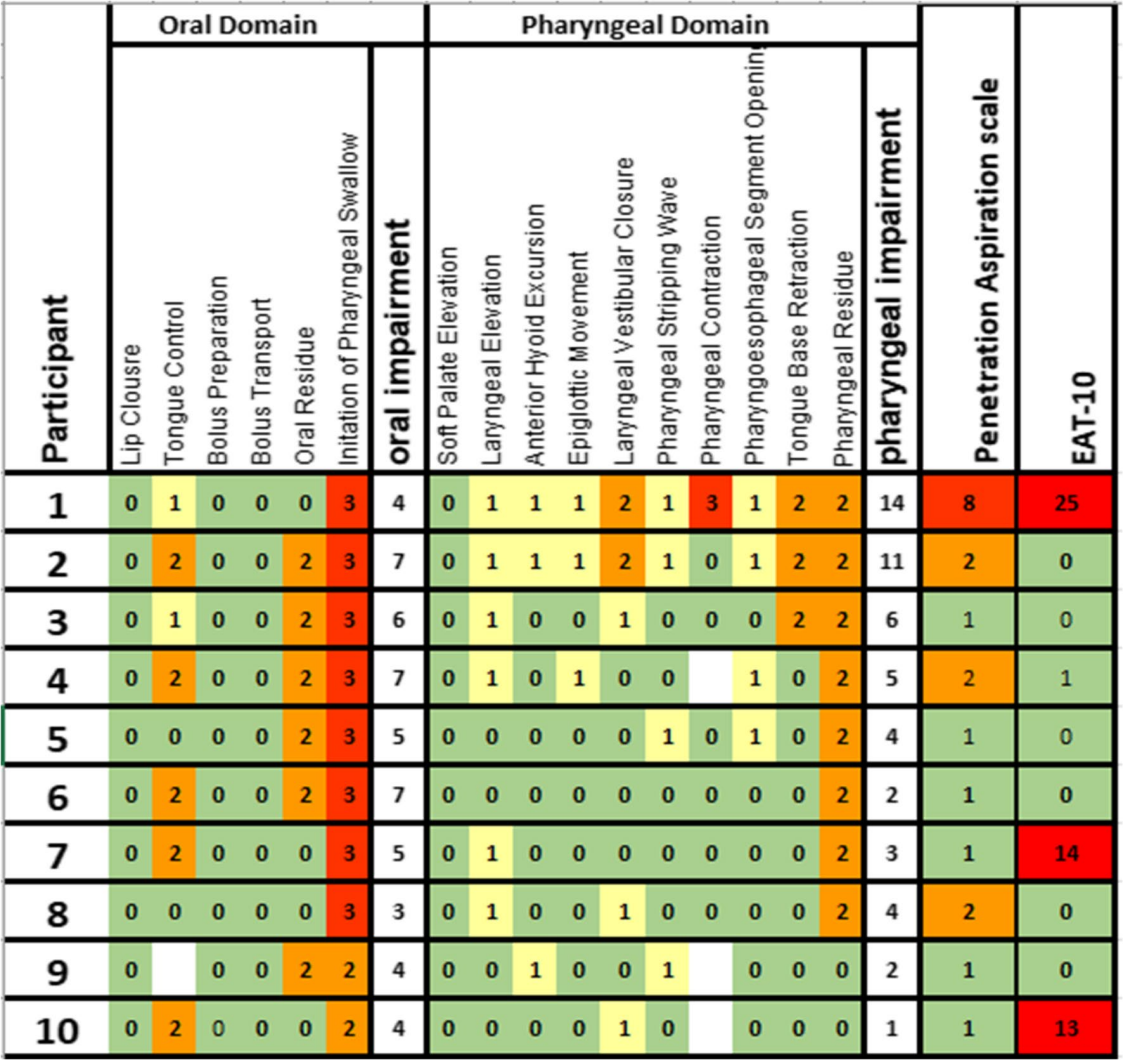
Videofluoroscopy Swallow Study (VFSS) and the Eating Assessment tool -10 results

Results for all patients relating to the 17 physiological components showing the Overall impression (OI) score, oral and pharyngeal impairment scores, PAS and EAT-10. EAT-10, Eating Assessment tool; Green cells indicate no dysfunction; yellow cells mild dysfunction; orange cells moderate dysfunction; red cells indicate severe dysfunction and white blank cells denote missing data

##### Swallow physiology: the MBS impairment profile

The *MBSImP* scores suggested none-to-mild swallowing impairment during oral and pharyngeal stages [16].

Lip closure, bolus preparation and transport, soft palate elevation were uniformly normal. However, all participants had evidence of oral residue, six had a reduction in tongue control and eight participants had a late initiation of pharyngeal swallow.

For the pharyngeal stage of swallowing five participants had evidence of incomplete laryngeal closure and one participant had a bilateral bulging of both pharyngeal walls. Eight participants had a reduction in tongue base retraction. Nine participants had evidence of post-swallow pharyngeal residue.

##### Swallow safety: the penetration/aspiration scale

**Fig. 3:**
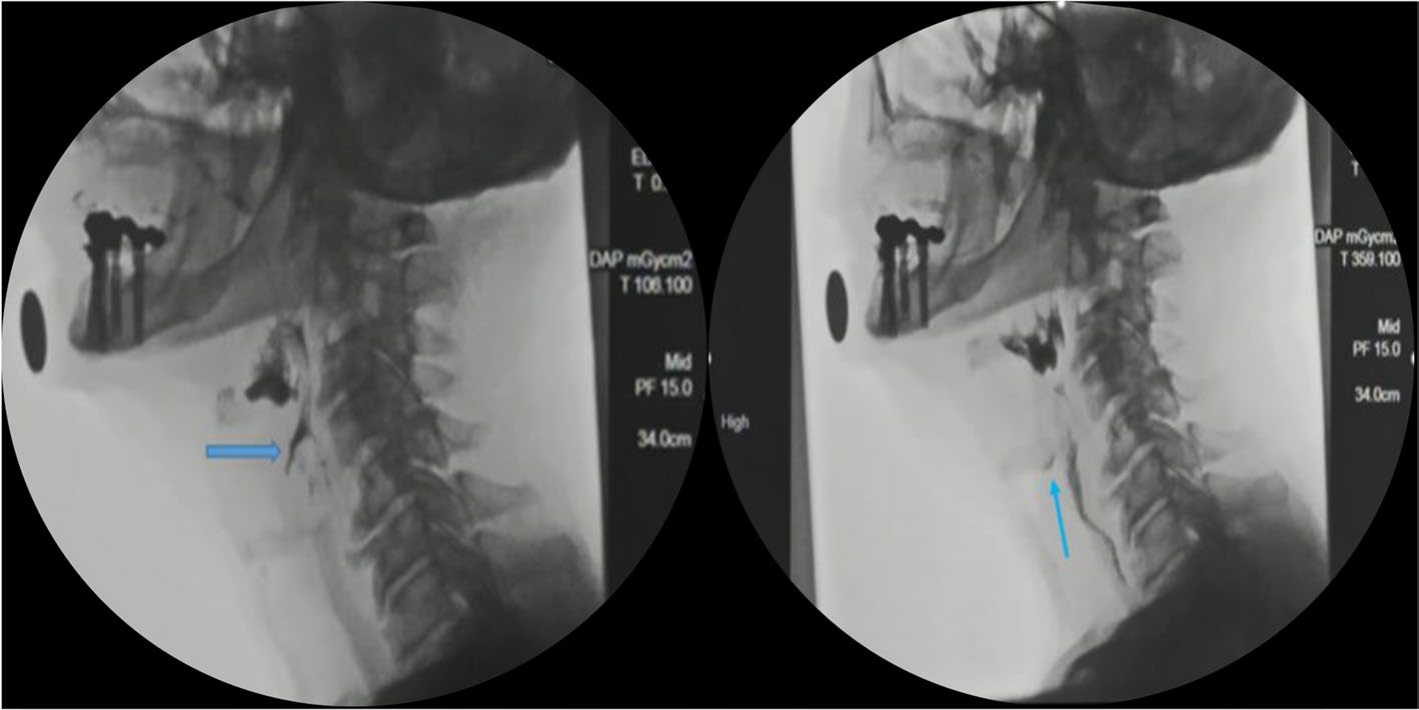
Oblique sagittal views during swallow of 20 mL thin liquid showing (left) penetration of barium into the larynx and (right) tracheal aspiration with contrast medium below the region of penetration

On PAS, 3/10 patients (Patients 2, 4 and 8) had airway penetration. Only patient 1 aspirated liquid without a cough response; this patient had reduced laryngeal elevation and incomplete laryngeal vestibular closure, resulting in the residue laying below the true vocal chords without a response to eject the aspirated liquid (Fig. 3).

## Discussion

To our knowledge, dysphagia has not been described in people with IPF. The purpose of this study was to describe oropharyngeal swallowing physiology and safety in unselected patients with a secure, mixed-disciplinary, diagnosis of IPF, without previous evidence for swallowing difficulty.

The widely used patient- reported symptoms of swallowing impairment, EAT-10 tool was used to assess patients’ perception of swallowing difficulty. In our study the median EAT-10 score was 0, range 0 -25. Four out of 14 patients (29%), had markedly raised total EAT-10 scores, with values of 25, 15, 14 and 13.

We are unaware of previous EAT-10 data in people with IPF. A recent study in 30 patients with Acute Exacerbations of COPD showed that 67% of patients had a raised EAT-10 score, compared with 23% of patients with cardiac disease [12].

In our study, subjective dysphagia symptoms reported by the EAT-10 tool were not consistently related to the nature and severity of the oropharyngeal swallow impairment observed during VFSS. Patient 1 who demonstrated aspiration on videofluorocopy had the highest EAT-10 score of 25, but patients who scored 13 and 14 in the EAT-10 tool had relatively normal swallow physiology detected during VFSS (Table: 2). Our exploratory findings in a limited number of patients are consistent with previous studies in COPD which have previously shown a weak association of EAT-10 with objective measurements of dysphagia [7, 12]. Further studies are therefore indicated in people with IPF, within which it is important to identify patients who may have difficulty swallowing regardless of whether aspiration is present. The EAT-10 tool helps to extend understanding about broad aspects of swallowing, which includes patient centred social and emotional information, not captured by objective instrumental tests.

Videofluoroscopy studies demonstrated a range of physiology in the ten patients studied. The swallow from laryngeal elevation onwards was consistently and highly disrupted in patient 1 and 2; these two patients had an abnormal physiology according to the *MBSImP* classification system [16]. However, patient 2 had no airway invasion, despite objectively the worst physiology of all, suggesting that a high score on *MBSImP* may not correspond to an unsafe swallow. Higher scores on some components of the *MBSImP* may be regarded as ‘normal’; for example, the bolus may enter the pharynx before swallow is initiated even in healthy individuals and some features may be part of a healthy ageing profile [20]. On PAS patients 2, 4 and 8 had airway penetration, we also noted that Patient 4 and 8 had relatively normal physiology by *MBSImP*. In the scarce literature, normal older swallowers sometimes have scores of 2 and 3 in PAS and our findings may therefore represent the combined effects of both normal aging and IPF pathophysiology and require further study [17]. There is no available evidence in the literature of which we are aware comparing the prevalence of swallowing impairments in IPF patients compared to the general population of similar age, with no known neurological disorders.

Parenchymal lung scarring and hypoxaemia may disrupt the complex coordination of normal swallowing and breathing function and in principle dysphagia may contribute to a complex dysregulated aerodigestive homeostasis in people with IPF. The true incidence of dysphagia in IPF is unknown but oral dysbiosis has been linked with a range of lung diseases including, pneumonia, COPD, and lung cancer [21]. The oral cavity has been shown to be a source of diverse bacteria and it is of interest that this can include non-gastric reservoirs of *Helicobacter pylori* [22], which has been associated with a more severe disease phenotype, higher mortality and lung function decline in people with IPF [23].

As fibrotic changes progress and lung function declines in IPF, the affected lung may be expected to become more susceptible to external challenges such as aspiration. Non-sterile aspiration, related to dysphagia and unprotected by cough, therefore represents a candidate source of complex lung injury and microbiome dysregulation. Aspiration is noted to be a trigger of threatening acute exacerbations [24]. Acute exacerbations in IPF are of high concern as they represent the most common cause of death in IPF. Just under a half of deaths in IPF are preceded by an acute exacerbation and the median survival after an acute exacerbation is approximately 3 to 4 months [25].

This prospective consecutive case series is descriptive in a limited number of patients. Further exploration is needed to establish the association between dysphagia and IPF, and the clinical significance of such a link. It would be of interest for further studies to assess if the prevalence of dysphagia in IPF is above that expected in a population of a similar age and to compare our IPF findings to other patient groups with comorbidities that could increase the risk of swallowing problems/aspiration (e.g. frailty). Our experience indicated that such studies are possible in people with IPF but also underline that these are challenging. Of 18 patients approached in our study five died, and three deaths occurred in fourteen consented patients, before videofluoroscopy could be performed. In other settings, simple bedside tests of dysfunction are clinically informative in swallowing pathophysiology [26] and together with selected patient reported outcome measures, [27] such approaches may be useful in frail patients. Safe approaches to augmented personalised therapy in selected patients, including speech and language intervention, could be rapidly implemented given the established model of mixed disciplinary care in IPF, if dysphagia is confirmed in further studies.

## Data Availability

The anonymised patient-level data used for this study cannot be shared for reasons of information governance. Data may be available to affiliated researchers given the North West- Preston Research Ethics committee, REC reference 14/NW/1056 ethical approval, and are available from the corresponding author on reasonable request.
